# TIPS improves outcomes in patients with HCC and symptomatic portal hypertension: a multi-institution experience

**DOI:** 10.1186/s40644-022-00451-9

**Published:** 2022-02-19

**Authors:** Huzheng Yan, Zhenkang Qiu, Zhanwang Xiang, Kai Feng, Mingsheng Huang, Fei Gao

**Affiliations:** 1 Department of Interventional Radiology, The Third Affiliated Hospital of Sun Yat-Sen University, 600 Tianhe Road, Guangzhou, 510630 China; 2 Department of Minimally Invasive & Interventional Radiology, State Key Laboratory of Oncology in South China, Collaborative Innovation Center for Cancer Medicine, Sun Yat-Sen University Cancer Center, 651 Dongfeng Road East, Guangzhou, 510060 China; 3 Department of Radiology, The Third People’s Hospital of Shenzhen, Shenzhen, China

**Keywords:** Hepatocellular carcinoma, Portal hypertension, Child–Pugh stage, TIPS

## Abstract

**Background:**

Hepatocellular carcinoma (HCC) with symptomatic portal hypertension (SPH) has poor prognosis. A transjugular intrahepatic portosystemic shunt (TIPS) relieves SPH, but its application in HCC remains unclear. We evaluated TIPS efficacy in patients with HCC and SPH.

**Methods:**

Pre- and post-TIPS Child–pugh(C–P) scores and stages in 123 HCC patients with SPH from three centers were compared. The impact of postoperative C–P stage indicators on overall survival (OS) was explored.

**Results:**

Post-TIPS responses to SPH included complete response (CR) (92 [74.8%]), partial response (PR) (23 [18.7%]), and nonresponse (NR) (8 [6.5%]). The control (proportion of CR and PR) for SPH was 93.5%. Median C–P scores pre-TIPS and at one month post-TIPS were 8 (IQR 6–9) and 7 (IQR 6–8), respectively (*P* < 0.001). Forty-one (33.3%) patients had C–P downstaging; 73 (59.3%) had lowered C–P scores; and 73 (59.3%) received intrahepatic local therapy post-TIPS. The median OS was 10.7 (1.1–55.2) months. Among the five indicators of C–P stage, lower post-TIPS ascites grading [(0/1)/(2/3); *P* = 0.014, HR = 0.31 (95% CI: 0.12–0.79)] and bilirubin [< 34/ ≥ 34 µmol/L; *P* = 0.022, HR = 0.47 (95% CI: 0.23–0.82)] and prothrombin time prolongation < 6 s [< 6/ ≥ 6 s; *P* = 0.001, HR = 0.17 (95% CI: 0.06–0.47)] were independent protective indicators of OS. These three indicators were included in the nomogram model to predict survival probabilities.

**Conclusions:**

TIPS is safe and effective for HCC with SPH. This procedure can relieve the symptoms, enable subsequent antitumor therapy, and bring survival benefits, possibly through improved liver function by reducing C–P stage.

## Introduction

Eighty percent of patients with hepatocellular carcinoma (HCC) have end-stage cirrhosis [1, 2]. Liver cirrhosis-related portal hypertension leads to serious complications such as refractory ascites and upper gastrointestinal bleeding, which exacerbate the liver function burden and lead to a poor prognosis [3, 4]. Most patients with HCC have symptomatic portal hypertension (SPH) [5, 6], which may disrupt tumor treatment and threaten survival prognosis [2, 3, 7].

The emergence of molecular-targeted drugs and immune checkpoint inhibitors has greatly improved survival rates of advanced HCC [8, 9]. However, the presence of SPH with HCC leads to more conservative treatment[2, 3]. Portal hypertension is an independent prognostic factor that increases 3- and 5-year mortality rates [10]. The European Association for the Study of the Liver recommends that patients with resectable HCC and clinically significant portal hypertension be evaluated circumspectly for appropriate treatment [2, 11, 12]. For patients with unresectable advanced HCC with SPH, there is currently no specific guideline to define the treatment strategy.

A transjugular intrahepatic portosystemic shunt (TIPS) has been recommended for managing SPH in end-stage liver disease [13]. Most studies have shown that TIPS can effectively control ascites, reduce the risk of esophagogastric vein rupture and re-bleeding, and improve liver function [3, 14]. The Child–Pugh (C–P) stage is most commonly used to evaluate liver function and prognosis in patients with cirrhosis. Its limitations have been described in detail [15]. For HCC patients with SPH, the liver function is more likely to be determined by the status of the tumor and the non-tumor liver. Specifically, the ascites and plasma albumin levels in the C–P stage are interrelated, and bleeding caused by portal hypertension seriously affects the C–P stage, while TIPS can improve the bleeding caused by portal hypertension. Therefore, we have to consider whether the C–P stage after TIPS represents the true prognosis of a patient. It is unknown whether the C–P stage is appropriate for HCC with SPH, and no research has clarified which of its five indicators is most likely to be improved after TIPS. With improved stent technology, some small studies have confirmed the feasibility and efficacy of TIPS in patients with HCC [16, 17]. However, little existing literature has reported overall survival (OS) related to TIPS in patients with HCC with SPH. The purpose of our study was to evaluate the efficacy of TIPS in HCC patients with SPH and to explore post-TIPS prognostic factors using an improved visualization nomogram model based on the C-P stage.

## Methods

### Study population

A total of 3911 patients with HCC from three interventional radiology centers from January 2016 to January 2020 were evaluated retrospectively. Inclusion criteria were as follows: diagnosed with HCC complicated by SPH, received TIPS, and had a tumor volume no more than 70% of the liver volume. Excluded patients had primary cholangiocarcinoma, multiple hepatic cysts, refractory biliary and pancreatic obstruction, liver failure, or severe cardiopulmonary dysfunction. Finally, 123 HCC patients with SPH who had received TIPS were included in this study. The Ethics Committee of the Sun Yat-sen University Cancer Center approved the study and waived the requirement for informed consent because as a retrospective study, this study did not interfere with patients’ treatment choices and thus was a very low risk to patients.

SPH in all of the patients manifested as refractory ascites (RA) (51 [41.5%]) and variceal bleeding (72 [58.5%]). Five cases (4.1%) had severe diarrhea. In terms of variceal bleeding, 15 (20.8%) patients had active variceal bleeding after endoscopic or medical treatment failure, and 57 (79.2%) patients underwent prevention for variceal re-bleeding.

### Definitions

The HCC diagnosis was based on the history of hepatitis, alpha-fetoprotein (AFP) levels, and imaging. SPH was defined as the presence of RA or variceal bleeding more than once. RA was classified into mild, moderate, or large/gross ascites in accordance with the International Club of Ascites definitions [3].

Responses to TIPS were classified as follows: complete response (CR), where no further variceal bleeding occurred and there was no clinically detectable ascites with or without a diuretic or salt-restricted diet; partial response (PR), where there was a small amount of ascites not requiring special paracentesis; and nonresponse (NR), where there was a large amount of ascites needing special intervention or there was recurrence of variceal bleeding [16].

Color Doppler ultrasonography (CDUS) was used to assess shunt patency. Shunt dysfunction was defined as a maximum flow velocity > 180 cm/s or < 60 cm/s in the shunt or < 30 cm/s in the main portal vein and a large amount of ascites or variceal re-bleeding. Suspected shunt dysfunction was confirmed with portal angiography showing a portosystemic pressure gradient (PPG) ≥ 15 mm Hg [16, 17].

### TIPS procedure

As previously described [16, 17], the TIPS procedure involved establishing an artificial shunt channel in the hepatic vein and the main portal vein or left or right branches through the jugular vein approach and implanting the shunt with a stent. All of the study patients received covered stent (Viatorr; W. L. Gore & Associates, Inc., Flagstaff, AZ, USA) implantation, and 27 (22.0%) received an additional bare stent (E-Luminexx; Bard Medical Division, Covington, GA, USA) implantation to ensure shunt function. The stent diameter was 8 mm, and the length was either 6, 8, or 10 cm. Portal and vena cava pressures were measured to calculate the PPG pre- and post-TIPS. Anticoagulant or diuretic treatments were administered post-TIPS as needed. Eighty-two (66.7%) patients received 10–20 mg/day oral rivaroxaban (Xarelto Fine Granules, Bayer, Leverkusen, Germany), and 74 (60.2%) patients received diuretic treatment or a salt-limited diet.

### Follow-up

All of the patients were followed up with until death or until the last contact, with primary end points being OS and effectiveness. The main observation indicators were C–P stage, C–P scores pre- and post-TIPS, responses to TIPS, and complications. The median follow-up time was 13 months. Laboratory tests of hematology, liver, kidney, and coagulation functions were performed every 1–2 weeks and then monthly. Abdominal CT/MR and CDUS were performed at weeks 1, 2, and 3 and then every two months. Examination intervals were shortened when symptoms recurred or as necessary.

### Statistical analysis

Statistical analysis was performed in SPSS software (IBM, Chicago, USA). All tests were two-tailed, and *P* < 0.05 was considered a significant difference. Continuous variables were presented using the median and interquartile range (IQR) values. We used the Pearson chi-square test to compare qualitative data and Student’s *t* test for quantitative data. Kaplan–Meier analysis (log-rank test) was used for subgroup survival analysis. The impact of the five indicators of postoperative C–P stage on OS was explored. The independent prognostic factors were selected to constitute the nomogram for prediction of survival probability.

## Results

### Patients’ characteristics pre-TIPS

A total of 123 patients were included in the study (Table 1). The median age was 58 (47–64) years, with 112 (91.1%) men and 11 (8.9%) women. A total of 117 (95.2%) patients had a history of hepatitis B. The median MELD was 14.5 (13.34–15.75). The distribution of Barcelona Clinic Liver Cancer (BCLC) classification stages A, B, C, and D was 14 (11.4%), 27 (22.0%), 71 (57.7%), and 11 (8.9%), respectively. Eighty-one (65.9%) patients had AFP levels ≥ 100 ng/mL; 81 (65.9%) had multiple tumors; 80 (65.0%) had portal vein tumor thrombosis (PVTT).

**Table 1:** Patient characteristics

**Characteristics**	***N* (%)/median (IQR**^a^**)**
Gender	
Male	112(91.1%)
Female	11(8.9%)
Age	58(47-64)
< 60	69(56.1%)
≥60	54(43.1%)
Aetiology of liver disease	
Hepatitis B	117(95.2%)
Hepatitis C	3(2.4%)
Alcoholic cirrhosis	3(2.4%)
BCLC classification^a^	
A	14(11.4%)
B	27(22.0%)
C	71(57.7%)
D	11(8.9%)
PVTT^a^	
No	43(35.0%)
Yes	80(65.0%)
Number of tumors	
Single	42(34.1%)
Multiple	81(65.9%)
MELD^a^	14.50(13.34-15.75)
<15	76(61.8%)
≥15	47(38.2%)
Symptomatic portal hypertension	
Refractory ascites	51(41.5%)
Variceal bleeding	49(39.8%)
Both	23(18.7%)
Refractory diarrhea	5(4.1%)
Laboratory tests	
Platelets [109/L]	88(65-125)
INR^a^	1.26(1.15-1.43)
AST [U/L] ^a^	46.3(34.0-65.0)
ALT [U/L] ^a^	37.6(25.4-52.1)
Albumin [g/dL]	33.2(30.2-36.8)
Creatinine [mg/dL]	72(62.2-86.5)
Bilirubin [mg/dL]	29.8(19.2-41.0)
AFP [ng/mL] ^a^	16.2(4.81-463.78)
<100	42(34.1%)
≥100	81(65.9%)

*PVTT* Portal vein tumor thrombosis, *MELD* Model of end stage liver disease, *INR* International normalised ratio, *AST* Aspartate aminotransferase, *ALT* Alanine transaminase, *AFP* Alpha fetoprotein, *CI* Confidence interval, *IQR* Interquartile range

^a^Barcelona Clinic Liver Cancer

### Antitumor therapy

One month post-TIPS, according to the SPH condition, liver function, and tumor stage, patients were reassessed with C–P stage and scores to make a decision about appropriate intrahepatic local treatment or systemic treatment. Intrahepatic local treatments included mainly transarterial chemoembolization (TACE) and microwave ablation (MWA), while systemic treatments included sorafenib (Xarelto Fine Granules, Bayer, Leverkusen, Germany), lenvatinib (Eisai Co Ltd, Tokyo, Japan), or apatinib (Hengrui Medicine, Jiangsu, China). Eighteen (14.6%) and 14 (11.4%) patients received TACE alone or MWA alone, respectively, and 41 (33.3%) patients received TACE combined with MWA. Among 36 (29.3%) patients who underwent systemic treatment, 11 (30.6%) received combined TACE or MWA treatment. Finally, 109 (88.6%) patients received antitumor therapy, and 73 (59.3%) received intrahepatic local therapy post-TIPS.

### Efficacy of TIPS and shunt dysfunction

The average PPG decreased from pre-TIPS (29.4 mm Hg) to post-TIPS (12.5 mm Hg). The pre-TIPS level of ascites was graded 0/I and II/III in 56 (45.5%) and 67 (54.5%) patients, respectively. At one month post-TIPS, ascites was graded 0/I and II/III in 107 (87.0%) and 16 (13.0%) patients, respectively. The number of patients with ascites graded II/III decreased (*P* < 0.001). Of the 72 patients with variceal bleeding as the main symptom, only seven (9.7%) reported this symptom one year post-TIPS. Overall, the responses of SPH to TIPS included CR (92 [74.8%]), PR (23 [18.7%]), and NR (8 [6.5%]). The control (proportion of CR and PR) for SPH was 93.5%.

Twenty (16.3%) patients had shunt dysfunction during the follow-up as confirmed by CDUS and enhanced CT. The primary patency rate at 90 days was 95.1% (115/123). The causes of shunt dysfunction included thrombosis (6 [30%]) and/or tumor invasion (14 [70%]), and 11 cases underwent a TIPS revision via balloon dilation or implantation of another bare stent. Nine patients refused further intervention and chose conservative treatment.

### TIPS-related complications

The most common post-TIPS complications were ALT/AST (77 [62.6%]) or bilirubin elevation (72 [58.2%]), which in most cases did not exceed four times the normal value. One case required percutaneous biliary drainage; one case developed multiple organ failure and died perioperatively; and the remaining patients recovered quickly after receiving liver protection treatment. Nine (7.3%) cases of suspected abdominal bleeding were monitored by continuous blood count and CDUS. Most of the patients recovered after conservative treatment (blood transfusion, hemostatic drugs, vital signs monitoring, etc.). Only one case developed hemorrhagic shock due to puncture injury rather than due to tumor rupture; this patient recovered after receiving interventional embolization for hemostasis and intensive care. Of the 13 (10.6%) patients who developed hepatic encephalopathy (HE) post-TIPS, only one had an HE of grade III/IV with loss of consciousness requiring intensive care treatment, while the remaining 12 patients had HE with grade I/II and received standard medical treatment to control HE.

### Changes in C–P Stages and five indicators

As shown in Table 2, pre-TIPS C–P stage A, B, and C was found in 32 (26.0%), 79 (64.2%), and 12 (9.8%) patients, respectively. One month after TIPS, there were 56 (45.5%), 58 (47.2%), and 9 (7.3%) patients with C–P stages A, B, and C, respectively. Forty-one (33.3%) patients had C–P downstaging, whereas in 70 (56.9%) and 12 (9.8%) patients, the C–P stage remained unchanged and increased, respectively. The post-TIPS C–P stage was lower (*P* = 0.06). The median preoperative and postoperative C–P scores were 8 (IQR 6–9) and 7 (IQR 6–8), respectively. Seventy-three (59.3%) patients had a lowered C–P score, and only 22 (17.9%) had an increased score; post-TIPS C–P scores were lower than pre-TIPS scores (*P* < 0.001).

**Table 2:** Changes in the Child–Pugh scores, Child–Pugh stages

**Variable**	**Before TIPS**	**One month after TIPS**	***P***
Response to TIPS^b^			
CR		92(74.8%)	
PR		23(18.7%)	
NR		8(6.5%)	
Child–Pugh stage			0.006*
A	32(26.0%)	56(45.5%)	
B	79(64.2%)	58(47.2%)	
C	12(9.8%)	9(7.3%)	
Change of Child–Pugh stage^b^			
Down		41(33.3%)	
Unchanged		70(56.9%)	
Elevated		12(9.8%)	
Child–Pugh scores	8(6-9)	7(6-8)	<0.001**
Change of Child–Pugh scores^b^			
Down		73(59.3%)	
Unchanged		28(22.8%)	
Elevated		22(17.9%)	
HE^a^			1.000*
I/II	4(3.3%)	12(9.8%)	
III/IV	0	1(0.8%)	
Grading of ascites			
0/1	56(45.5%)	107(87.0%)	<0.001*
2/3	67(54.5%)	16(13.0%)	
Bilirubin(IQR^a^, umol/L)	29.5(19.2-41.0)	37.4(25.6-48.1)	<0.001**
<34	75(61.0%)	51(41.4%)	
≥34	48(39.0%)	72(58.6%)	
Albumin (g/L)	33.2(30.2-36.8)	34.7(33.1-36.8)	0.134**
<35	72(58.5%)	65(52.8%)	
≥35	51(41.5%)	58(47.2%)	
PT(s)^a^	14.2(13.1-15.6)	15.5(14.1-17.1)	<0.001**
Prolonged < 6	117(95.1%)	71(57.7%)	
Prolonged ≥6	6(4.9%)	52(42.3%)	

^a^*HE* Hepatic encephalopathy, *PT* prothrombin time; ^b^One month after TIPS, the Child-Pugh stage and Child-Pugh score were reassessed. The responses to TIPS: complete response (*CR*), no further variceal bleeding and having no clinically detectable ascites with or without diuretic or salt-restricted diet; partial response (*PR*), having a small amount of ascites not requiring special paracentesis; and nonresponse (*NR*), having a large amount of ascites needing special intervention or variceal bleeding recurrence. *Chi-square test; **Paired t-test

The incidence of HE post-TIPS (13 [10.6%]) was not significantly higher than that of pre-TIPS (4 [3.3%]) (*P* = 1.000). The median pre- and post-TIPS bilirubin level was 29.5 µmol/L (IQR,19.2–41.0) and 37.4 µmol/L (IQR, 25.6–48.1), respectively, and prothrombin time (PT) was 14.2 s (IQR,13.1–15.6) and 15.5 s (IQR, 14.1–17.1), respectively. Post-TIPS bilirubin and PT increased (*P* < 0.001). The pre- and post-TIPS grades 2 and 3 of ascites were found in 67 (54.5%) and 16 (13.0%) patients, respectively. Post-TIPS ascites was improved (*P* < 0.001), and albumin levels (34.7 g/L [33.1–36.8]) had an increasing trend (*P* = 0.134) compared with pre-TIPS levels (33.2 g/L [30.2–36.8]).

## OS

The median OS was 10.7 (range, 1.1–55.2) months. The median OS of BCLC A, B, C, and D was 18 (range, 2.6–51.3), 15.1 (range, 1.6–55.2), 7.8 (range, 1 –39.1), and 5.3 (range, 3.6–30.5) months, respectively. The OS of HCC with RA and variceal bleeding was 10.3 (range, 1.1–51.3) and 10.8 (range, 1.2–55.2) months, respectively.

**Fig. 1:**
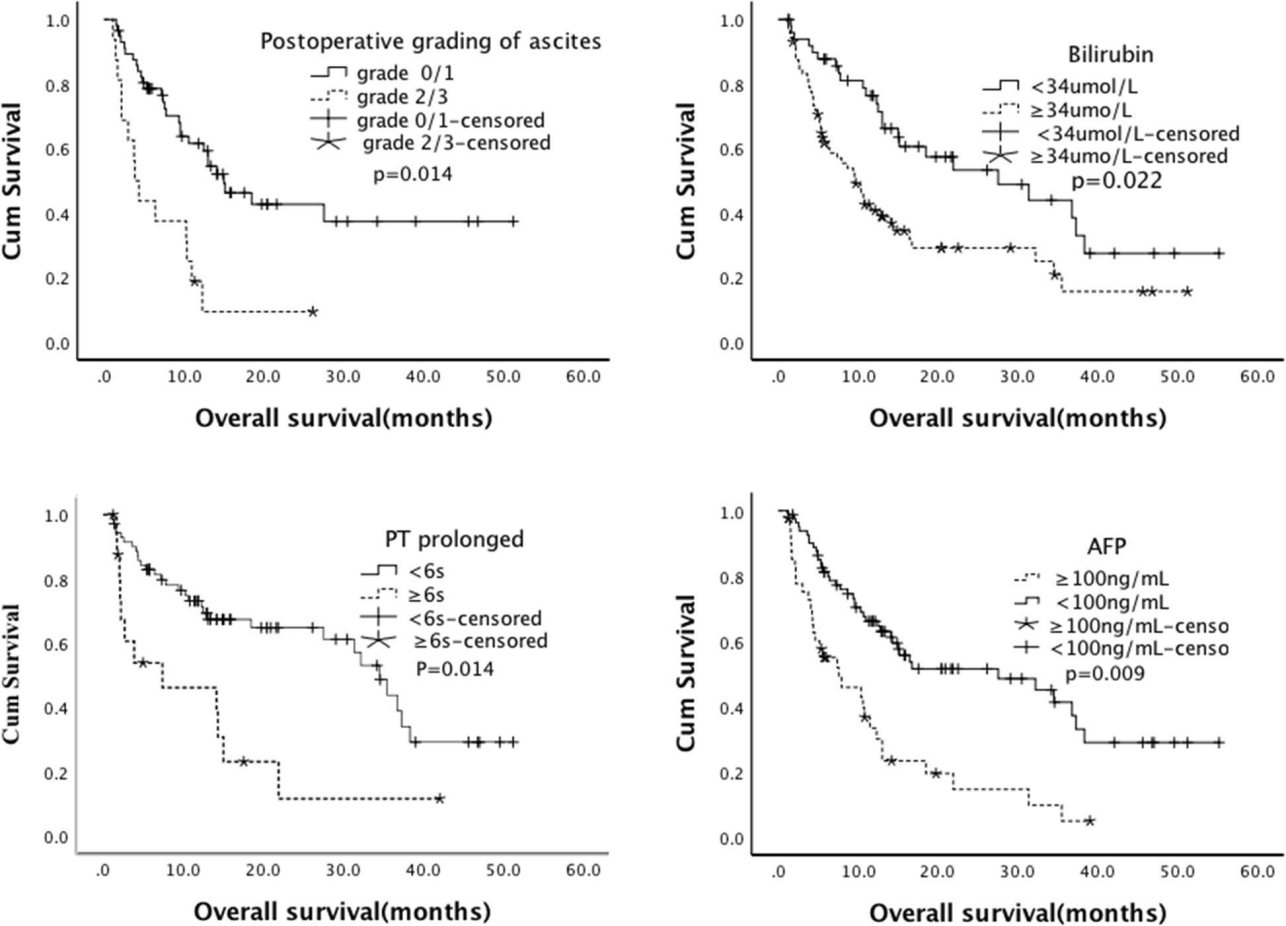
Overall survival (Kaplan–Meier analysis). **a** Postoperative Child–Pugh stage. **b** Bilirubin. **c** Prolonged prothrombin time (PT). **d** Alpha-fetoprotein (AFP)

As shown in Table 3 and Fig. 1, pre-TIPS, patients in C–P stage A had a longer OS than those in stage C [A/C: *P* = 0.005, HR = 0.27 (95% CI: 0.11–0.67)], and there was no difference in OS between stages B and C [B/C: *P* = 0.335, HR = 0.69 (95% CI: 0.32–1.46)]. One month post-TIPS, cases with a lower C–P stage [A/C: *P* < 0.001, HR = 0.13 (95% CI: 0.06–0.28); B/C: *P* < 0.001, HR = 0.22 (95% CI: 0.10–0.47)] or a C–P stage that did not increase [non-elevated /elevated: *P* = 0.003, HR = 0.37 (95% CI: 0.20–0.71)] showed better OS. The CR-to-TIPS [CR/(PR/NR): *P* < 0.001, HR = 0.38 (95% CI: 0.23–0.63)] also showed better OS.

**Table 3:**
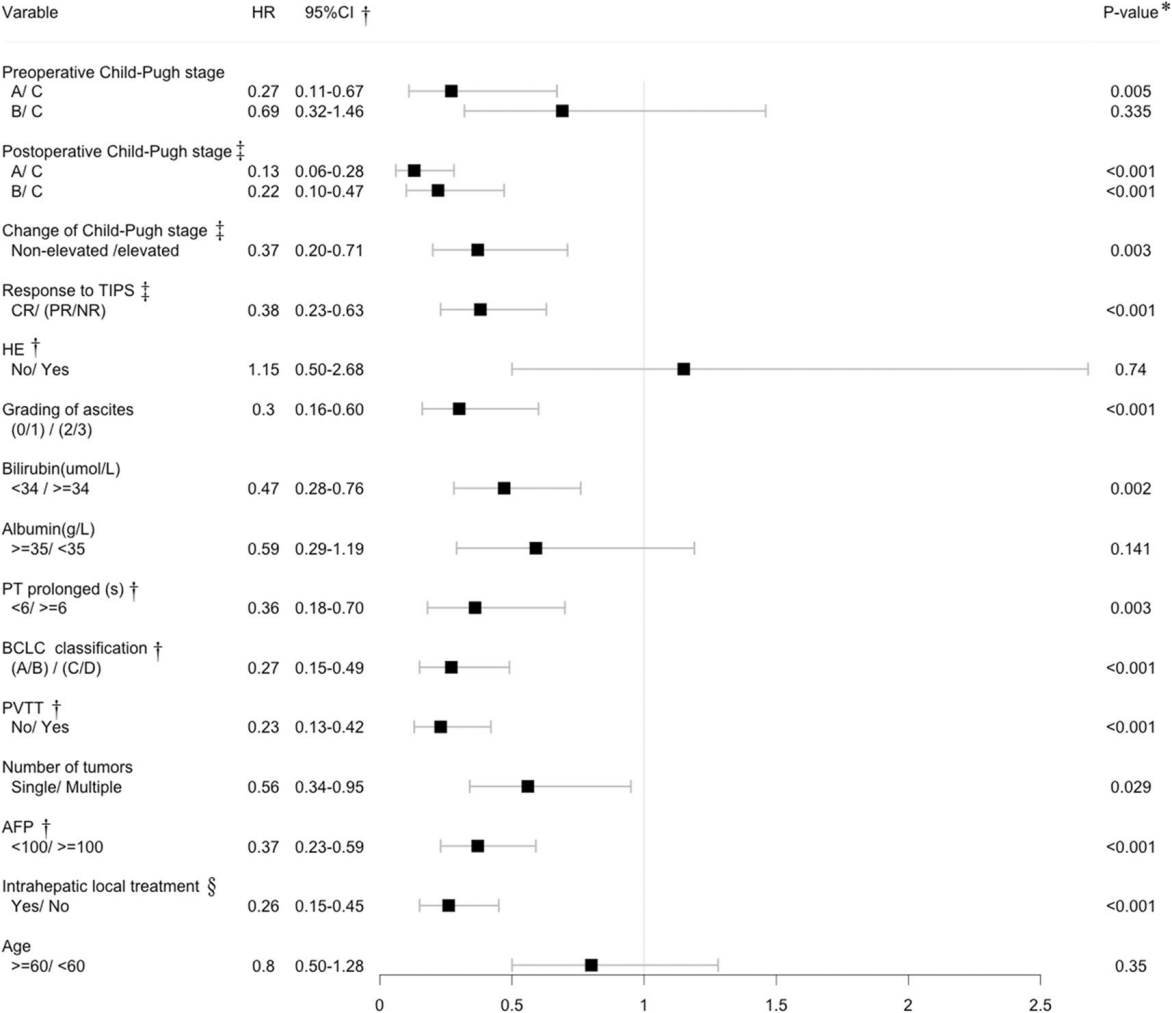
Univariate analysis related to OS

†*HE* Hepatic encephalopathy, *PT* Prothrombin time, *BCLC* Barcelona Clinic Liver Cancer, *PVTT* Portal vein tumor thrombosis, *AFP* Alpha fetoprotein, *CI* Confidence interval; ‡One month after TIPS, the Child-Pugh stage and Child-Pugh score were reassessed. The responses to TIPS: complete response (*CR*), no further variceal bleeding and having no clinically detectable ascites with or without diuretic or salt-restricted diet; partial response (*PR*), having a small amount of ascites not requiring special paracentesis; and nonresponse (*NR*), having a large amount of ascites needing special intervention or variceal bleeding recurrence. §Including transarterial chemoembolization and ablation. P* Chi-square test

Among the five indicators of post-TIPS C–P stage, good OS was related to lower grading of ascites [(0/1)/(2/3): *P* < 0.001, HR = 0.30 (95% CI: 0.16–0.60)], lower level of bilirubin [< 34/ ≥ 34 µmol/L: *P* = 0.002, HR = 0.47 (95% CI: 0.28–0.76)], and PT prolonged for < 6 s [< 6/ ≥ 6 s: *P* = 0.003, HR = 0.36 (95% CI: 0.18–0.70)]. There was no significant difference in HE or level of albumin ≥ 35 or < 35 g/L (*P* > 0.05). In terms of tumor factors, good OS results were associated with a lower BCLC classification, no PVTT, presence of a single tumor, lower level of AFP, and intrahepatic local treatment.

Table 4 shows the Cox proportional-hazards regression analysis, indicating lower postoperative grade of ascites [(0/1)/(2/3): *P* = 0.014, HR = 0.31 (95% CI: 0.12–0.79)], lower postoperative level of bilirubin [< 34/ ≥ 34 µmol/L: *P* = 0.022, HR = 0.47 (95% CI: 0.23–0.82)], prolonged post-TIPS PT < 6 s [< 6/ ≥ 6 s: *P* = 0.001, HR = 0.17 (95% CI: 0.06–0.47)], and AFP level < 100 ng/mL [< 100/ ≥ 100 ng/mL: *P* = 0.009, HR = 0.31 (95% CI: 0.13–0.75)] as the independent predictors of better OS.

**Table 4:**
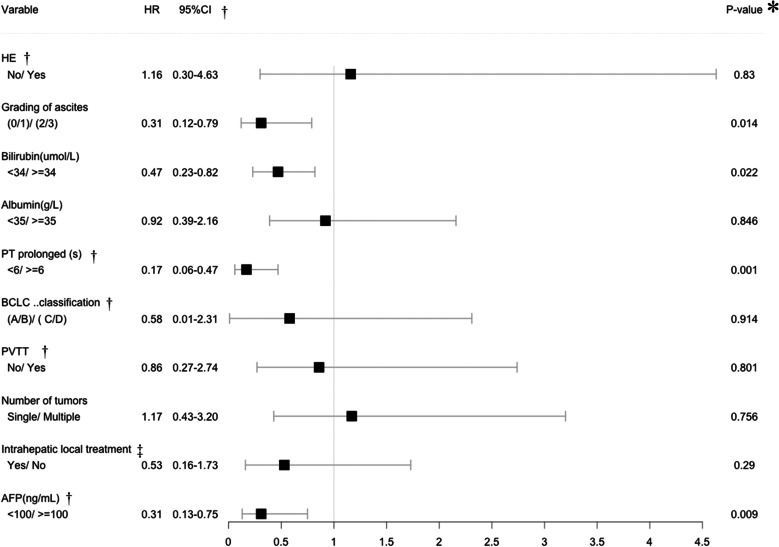
Cox proportional hazards regression analysis related to OS

†*HE* Hepatic encephalopathy, *PT* Prothrombin time, *BCLC* Barcelona Clinic Liver Cancer, *PVTT* Portal vein tumor thrombosis, *AFP* Alpha fetoprotein, *CI* Confidence interval; ‡.§Including transarterial chemoembolization and ablation. P* Chi-square test

**Fig. 2:**
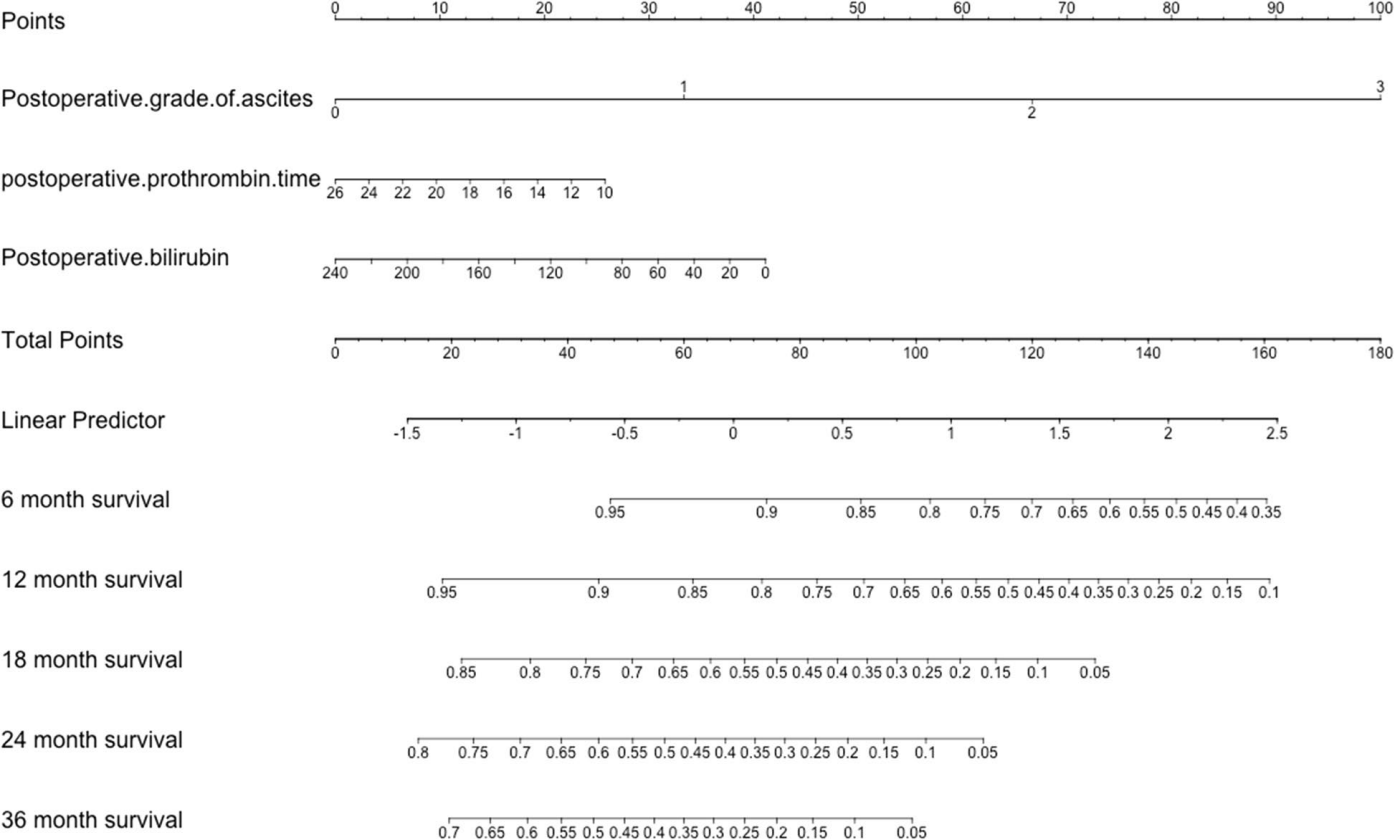
Nomogram for predicting the probability of overall survival. A simple model based on the Child–Pugh stage was used to visually predict the overall survival probability. This model includes postoperative grading of ascites, postoperative level of bilirubin, and prothrombin time (PT). The total scores of the three indicators correspond to the predicted survival probabilities at 6, 12, 18, 24, and 36 months

Based on the statistically significant variables from the multivariate analysis, as shown in Fig. 2, a nomogram was constructed based on the above three variables to predict survival probabilities at 6, 12, 18, 24, and 36 months.

## Discussion

SPH severely affects the prognosis of patients with HCC and increases the risk of death. Our research demonstrated that TIPS can bring survival benefits, which may occur through the improved liver function. We also proposed a simple model to predict OS based on the improved C–P stage.

There are no treatment guidelines for HCC with SPH. The few reports on the use of TIPS in HCC have described its feasibility and safety [16–19]. The OS of HCC is determined by tumor burden factors, liver function, and complications related to portal hypertension [2, 3, 7]. Complications associated with SPH may be more life-threatening than the tumor burden because SPH often leads to emergency situations [13, 17, 20, 21]. Our results showed that control (proportion of CR and PR) of SPH was achieved in 93.5% (115/123) of patients, and only 6.5% (8/123) of patients showed NR; thus, TIPS was effective for patients with HCC. This high control rate indicates that TIPS can alleviate the symptoms of SPH in patients with HCC and reduce the associated risk of death. This is the largest study to date, comprising 123 cases, demonstrating the effectiveness and feasibility of TIPS in patients with HCC and proposing a post-TIPS prognostic evaluation model.

**Fig. 3:**
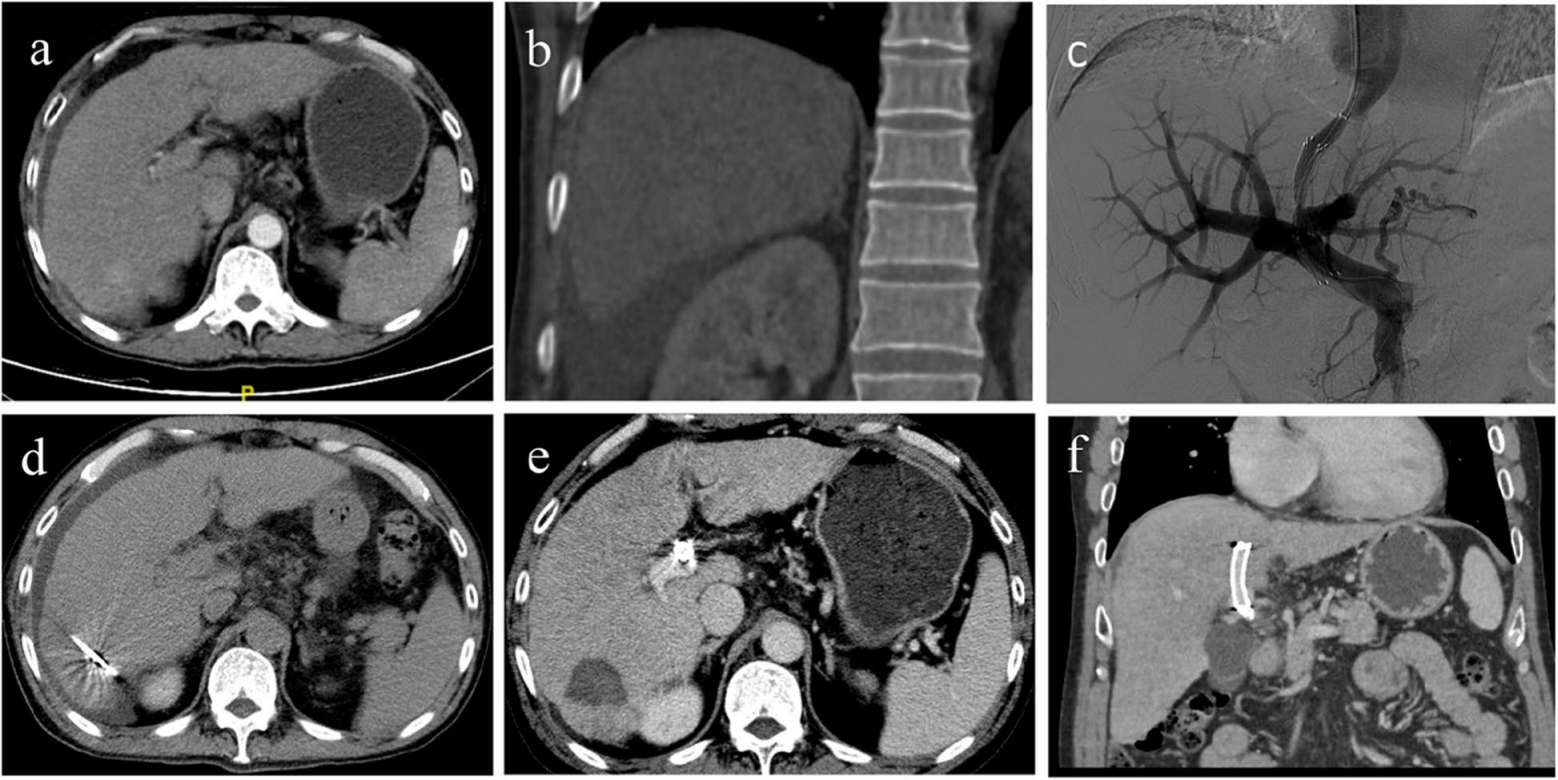
Case of TIPS. A 53-year-old male patient with a primary hepatocellular carcinoma (HCC) with a diameter of 3 cm. He had experienced a repeated diuretic therapy because of his refractory ascites. **a**, **b** Ascites and tumor before transjugular intrahepatic portosystemic shunt (TIPS). The Child–Pugh score and Child–Pugh stage were 9 and (B), respectively. **c** TIPS was completed. **d** Two weeks after TIPS, a microwave ablation treatment was implemented. **e**, **f** One month after TIPS, the reevaluated Child–Pugh score and Child–Pugh stage were 7 and (B) respectively. The overall survival of the patient exceeded four years, and he is still alive

The median OS in our study of 10.7 (range, 1.1–55.2) months, 10.3 (range, 1.1–51.3) months in patients with RA, and 10.8 (range, 1.2–55.2) months in patients with variceal bleeding is in disagreement with previous studies showing a 6-month median OS of RA in end-stage liver disease [3]. Liu et al. reported 77 days as a median OS post-TIPS in HCC with SPH [22]. The survival of these enrolled patients, considering tumor factors, was better than that in previous studies, especially in BCLC classification C (median 7.8 months) and D (median 5.3 months) [3, 13, 17, 18, 22, 23]. This may be related to the following: (1) Three months post-TIPS, the RA control was 90.5% (Fig. 3), and only 1.4% (1/72) of patients had re-bleeding; (2) 41 (33.3%) patients had downgraded C–P staging and greatly improved liver function; 3) 73 (59.3%) patients received intrahepatic local treatment, which may have reduced the liver tumor burden.

Child–Pugh staging is one of the most widely used liver function assessments, but its appropriateness for use in HCC is unclear, especially in patients with SPH [15, 24, 25]. Ascites and hypoalbuminemia may be caused by SPH. A different liver function evaluation is needed for these patients post-TIPS. By comparing the five indicators of Child–Pugh staging pre-TIPS and one month post-TIPS, we found that PT, bilirubin, and HE had increased and that 41 (33.3%) patients had a lowered C–P stage post-TIPS. The post-TIPS albumin level increased slightly (median: 34.7 versus 33.2 g/l) (*P* = 0.134). Post-TIPS grade 2/3 ascites was reduced from 54.5% to 13.0% (*P* < 0.001). Therefore, improvement of albumin and ascites improved the C–P stage. Our study further used five indicators of C–P stage to explore the impact of TIPS on OS. The univariate analysis and multivariate analysis found differences in PT, bilirubin, and grade of ascites. The Cox proportional-hazards regression analysis showed that lower post-TIPS grade of ascites [(0/1)/(2/3): *P* = 0.014], lower post-TIPS level of bilirubin [< 34/ ≥ 34 µmol/L: *P* = 0.022], and post-TIPS PT prolonged < 6 s [< 6/ ≥ 6 s: *P* = 0.001] were independent predictors of better OS. There was no difference in albumin level [< 35/ ≥ 35 g/L: *P* = 0.846], possibly because hypoalbuminemia and ascites are interrelated indicators and influence one another, and that albumin supplementation therapy may lead to an increase in albumin levels (and thus, an improvement in C-P stage), but may not accurately reflect the liver’s ability to synthesize albumin. Based on the above shortcomings, we developed the nomogram prediction system comprising the three indicators of post-TIPS, PT, bilirubin, and grade of ascites to replace the C–P system. This model uses grading to reflect the true liver function in HCC with SPH.

There has been no guideline for the management of HCC with SPH. SPH affects HCC treatment strategy, and HCC makes the treatment of SPH more conservative. Therefore, our research provided some data for the management of these patients. Our research also reflected that a better C–P score after TIPS seems to result in a longer OS. We explained this result on the basis of the improvement of liver function and subsequent antitumor therapy activity. Therefore, the question arises whether earlier TIPS may bring survival benefits for HCC with SPH, just as early TIPS for high-risk variceal bleeding obviously improves the 6-week re-bleeding rate and 1-year survival compared with endoscopic treatment [26]. One key reason is that early TIPS reduces the impact of re-bleeding on the liver function of patients with end-stage liver disease [27]. However, in HCC patients with SPH, liver function suffers from bleeding and refractory ascites, which severely affects the prognosis and also prevents some patients from entering the subsequent antitumor treatment. Our study showed a more aggressive TIPS strategy in HCC with SPH to improve liver function so that HCC patients can tolerate subsequent antitumor treatments and obtain survival benefits. This benefit also needs to be further confirmed by more large-scale prospective studies.

Our study also explored the impact of tumor burden factors on OS. Good OS was associated with a lower BCLC classification (*P* < 0.001), no PVTT (*P* < 0.001), a single tumor (*P* = 0.029), a lower level of AFP (*P* < 0.001), and intrahepatic local treatment (*P*< 0.001). AFP is an independent prognostic factor, and the prognostic factors identified are similar to those identified in previous studies. We showed that the prognosis of HCC with SPH was determined by multiple factors, including tumor burden [15, 28–30].

Our study showed a technical success rate of 98.4% (121/123). Two patients (1.6%) achieved success for the second time, which was a higher result than that reported in a previous study [31]. Although 65% of the patients in our study had PVTT, most of the patients with tumor thrombus did not have secondary portal cavernous evolution. Technically, it is not too difficult compared to regular TIPS, and only three patients with severe portal vein cavernosis required percutaneous liver puncture and venography to complete TIPS. Although PVTT is a relative contraindication for TIPS, with the emergence of new stent-grafts, the long-term shunt patency in patients with PVTT can be improved. Moreover, TIPS can quickly relieve the symptoms of portal hypertension caused by PVTT, improve opportunities for subsequent targeted therapy, and also help control tumor thrombus. This treatment modality deserves further exploration of its benefits in patients with PVTT. One (0.8%) patient required interventional embolization for intraabdominal hemorrhage. Our study identified fewer adverse events than the study by Liu et al., [22] who reported the incidence of tumor rupture of 8.6% in 58 patients, which may have been related to PVTT in all of their patients. The incidence of HE in our study was 10.6%, lower than the rate of 44% reported by Wallace et al., [32] which may be related to the 8 mm diameter stents we used.

Our research has a few limitations. The median follow-up time of patients with the BCLC classifications of A and B was only 12.5 months, and 50% of these patients were still living, thus reducing the OS of these patients. The nomogram prediction based on retrospective research and a sample of 123 cases requires more cases to verify our results.

## Conclusion

TIPS is safe and effective for HCC with SPH. This procedure can relieve the symptoms, enable subsequent antitumor therapy, and bring survival benefits, which may come from the improved liver function from the reduced C–P stage.

## Data Availability

The datasets used and/or analysed during the current study are available from the corresponding author on reasonable request.

## Abbreviations

HCC: Hepatocellular carcinoma
SPH: Symptomatic portal hypertension
TIPS: Transjugular intrahepatic portosystemic shunt
C–P: Child–Pugh
RA: Refractory ascites
PPG: Portosystemic pressure gradient
CDUS: Color doppler ultrasonography
CR: Complete response
PR: Partial response
NR: None response
TACE: Transarterial chemoembolization
MWA: Microwave ablation
OS: Overall survival
AFP: Alpha-fetoprotein
PVTT: Portal vein tumor thrombosis
MELD: Model of end-stage liver disease
IQR: Interquartile range
